# Coronary artery disease and neurocognitive performance among older adults in Uganda

**DOI:** 10.21203/rs.3.rs-10737115/v1

**Published:** 2026-09-10

**Authors:** Richard Migisha, Jolina Lombardi, Pauline Byakika-Kibwika, Noeline Nakasujja, Samuel Maling, Peter Kangwagye, Abraham Muhwezi, Christopher T. Longenecker, Samson Okello, Moses Acan, Flavia Atwiine, Edna Tindimwebwa, Zahra Reynolds, Robert Paul, Brian Ghoshhajra, Eliza Passell, Deanna Saylor, Miria Kyokusiima, Geoffrey Erem, Alexander C. Tsai, Jeremy A. Tanner, Stephen Asiimwe, Mark J. Siedner

**Affiliations:** Mbarara University of Science and Technology; University of California, San Francisco; Mbarara University of Science and Technology; Makerere University; Mbarara University of Science and Technology; Mbarara University of Science and Technology; Mbarara University of Science and Technology; University of Washington; University of North Carolina at Chapel Hill; Mbarara University of Science and Technology; Mbarara University of Science and Technology; Kabwohe Clinical Research Center; Massachusetts General Hospital; University of Missouri-St. Louis; Massachusetts General Hospital; Massachusetts General Hospital; University of North Carolina at Chapel Hill; Mbarara University of Science and Technology; Makerere University; Massachusetts General Hospital; University of Texas Health Science Center at San Antonio; Mbarara University of Science and Technology; Mbarara University of Science and Technology

**Keywords:** Coronary artery disease, cognitive function, neurocognitive performance, HIV, ageing, Uganda

## Abstract

**Background:**

Coronary artery disease (CAD) has been linked with poorer cognitive function, potentially through shared risk factors and vascular pathways linking atherosclerotic disease to cerebral vascular injury. However, the association between CAD and neurocognitive performance remains poorly characterized in African populations.

**Methods:**

We analyzed data from the Uganda Aging and Dementia Cohort Study among participants with coronary computed tomography imaging and neurocognitive testing data. The primary exposure was imaging-defined CAD, and cognitive performance was evaluated at the global-composite, domain-composite, and individual-test levels. Multivariable linear regression models adjusted for age, sex, and HIV status. HIV-stratified models adjusted for age and sex, and CAD-by-HIV interaction terms assessed effect modification. We examined predicted 10-year atherosclerotic cardiovascular disease (ASCVD) risk > 5% as an alternative cardiovascular-risk exposure. Higher b coefficients indicated poorer performance. Benjamini-Hochberg false-discovery-rate (FDR) correction was applied within outcome tiers.

**Results:**

Among 512 participants, mean age was 57.9 years (SD, 6.3), 252 (49.2%) were female, 238 (46.5%) were people with HIV (PWH), 42 (8.2%) had CAD, and 203 (39.6%) had ASCVD risk > 5%. CAD was not associated with global cognition (b = 0.093 SD units; 95% CI, – 0.122 to 0.308; P = 0.397). At individual-test level, CAD was associated with poorer performance on Color Trails 2 (a measure of executive function) (b = 0.502; 95% CI, 0.184 to 0.821; P = 0.002; q = 0.026) and nominally associated with poorer performance on Digits Forward (a measure of attention and immediate verbal span) (b = 0.386; 95% CI, 0.028 to 0.744; P = 0.035; q = 0.210). In HIV-stratified analyses, CAD was nominally associated with poorer Digits Forward performance among PWOH (b = 0.552; P = 0.006; q = 0.070) and poorer Color Trails 2 performance among PWH (b = 0.646; P = 0.010; q = 0.121), but neither survived FDR correction and interaction tests did not support effect modification. ASCVD risk > 5% was nominally associated with poorer learning-and-memory domain performance (b = 0.188; 95% CI, 0.026 to 0.350; P = 0.023; q = 0.116) and poorer WHO/UCLA AVLT total recall (b = 0.231; 95% CI, 0.048 to 0.414; P = 0.014; q = 0.162); neither survived FDR correction.

**Conclusion:**

CAD was not associated with global or domain-level neurocognitive performance. Its association with poorer Color Trails 2 performance may suggest selective vulnerability in executive control, but requires confirmation in larger longitudinal studies.

## Introduction

Coronary artery disease (CAD) and cognitive decline are major age-related conditions with overlapping vascular and inflammatory pathways (1). Among older adults in the global North, coronary heart disease has been associated with higher risk of cognitive impairment or dementia, with meta-analytic evidence suggesting approximately 45% higher odds of subsequent cognitive impairment among persons with coronary heart disease (1). Cognitive deficits in patients with CAD may reflect cerebral hypoperfusion, impaired cerebrovascular reactivity, endothelial dysfunction, microvascular injury, systemic inflammation, and shared cardiometabolic risk factors (2, 3). Because vascular brain injury often affects frontal–subcortical circuitry, CAD-related cognitive changes may be more evident in processing speed, attention, and executive control than in global cognition or all domains uniformly (4, 5). Domain- and test-level measures may therefore be particularly informative for detecting selective neurocognitive vulnerabilities associated with CAD. Yet, there are limited data on CAD–cognition association in sub-Saharan Africa where the population is rapidly aging and the burden of cognitive impairment is projected to rise (6, 7).

People with HIV (PWH) on antiretroviral therapy may be at particular risk for both vascular disease and neurocognitive impairment (8, 9), due to a combination of traditional cardiometabolic risk factors, direct viral pathology, persistent immune activation, endothelial dysfunction, and chronic inflammation despite viral suppression (10, 11). Vascular risk factors and cardiovascular disease have also been associated with worse cognitive performance and neurocognitive impairment among PWH in the era of combination antiretroviral therapy (12). In many areas of sub-Saharan Africa, where the HIV prevalence remains high and the population of older PWH is growing, most studies have focused on HIV-associated neurocognitive disorders without detailed evaluation of objectively measured vascular disease (13, 14). Evidence from Uganda suggests that CAD epidemiology among older PWH and people without HIV (PWOH) may differ from that in high-income settings, underscoring the need for locally generated evidence (15, 16).

Despite biological plausibility, few studies have directly examined whether CAD is associated with cognitive functioning among older PWH and PWOH in rural sub-Saharan Africa. Vascular cognitive impairment may be preventable or modifiable through cardiovascular risk reduction, earlier detection of coronary disease, and integration of cognitive screening into chronic HIV and non-communicable disease care. Using data from a longitudinal cohort study of older adults in southwestern Uganda, we assessed the association between imaging-defined CAD and neurocognitive performance across global, domain-level, and individual-test outcomes. In secondary analyses, we examined associations between high predicted atherosclerotic cardiovascular disease (ASCVD) risk and neurocognitive performance.

## Methods

### Study design, setting and population

We conducted an analysis of data from older adults (aged > 40 years) enrolled in the Uganda Aging and Dementia Cohort Study (17) and the affiliated Epidemiology of Coronary Artery Disease in Uganda study in southwestern Uganda (16). The parent studies enrolled a shared cohort of people with HIV (PWH) on ART for a minimum of three years, and age- and sex-similar, population-based participants without HIV (PWOH) enrolled from the same communities.

### Study participants

Participants were followed annually and underwent standardized clinical and neurocognitive assessments at each study visit. Cohort participants were invited to participate in a cardiovascular disease sub-study, which included coronary computed tomography angiography and coronary artery calcium scoring (16). For the present analysis, we included participants with a valid global cognitive composite score and at least one normalized individual test score contributing to each of the prespecified non-motor domains most relevant to vascular cognitive vulnerability: attention/processing speed, learning/memory, and executive function/working memory. Because coronary imaging and neurocognitive testing were not performed on the same day, each imaging assessment was paired with the neurocognitive assessment from the closest annual cohort visit, which occurred within 12 months of CAD imaging. Participants with missing data for an outcome or covariate required for a specific model were excluded from that model only. To assess potential selection bias from complete-case modelling, we compared characteristics of participants included in the primary multivariable CAD model with those not included because of missing outcome, exposure, or covariate data.

### Coronary artery disease assessments

The primary exposure was imaging-defined CAD, categorized as present or absent. Full details of the scanning procedures and image interpretation have been reported previously (16). In brief, CAD was defined as either calcified or noncalcified plaque identified on coronary computed tomography angiography in at least one major coronary artery or branch, or an Agatston coronary artery calcium score greater than 0 (18). Coronary images in the parent study were interpreted by cardiovascular imaging specialists who were blinded to participants’ HIV status and clinical characteristics.

### Neurocognitive assessments

Neurocognitive testing followed the Uganda Aging and Dementia Cohort Study cognitive protocol(17). Participants completed the Runyankore-language Uganda Neuropsychological Battery under the administration of trained research staff. The battery included cross-culturally adapted tests previously used in Ugandan HIV neurocognitive studies (19-21).

The battery assessed five cognitive domains. Attention and processing speed were assessed using Color Trails 1 and Digits Forward. Learning and memory were assessed using total recall and 30-minute delayed recall scores from the World Health Organization–University of California, Los Angeles Auditory Verbal Learning Test. Executive functions and working memory were assessed using Symbol Digit, Color Trails 2 and Backward Digit Span. Language was assessed using animal fluency. Motor function was assessed using timed gait, finger tapping, and Grooved Pegboard tests, with pegboard performance measured separately for the participant’s dominant and non-dominant hands. Additional details on test administration and score construction are provided in Supplementary **Table S1**.

### Neurocognitive performance outcomes

Neurocognitive outcomes were continuous normalized scores. The global cognitive composite was the primary outcome, domain-specific composites and individual-test scores were secondary outcomes. We examined a global cognitive composite score, five domain-specific composite scores—attention, learning and memory, executive function, language, and motor function—and normalized individual test scores. The global cognitive composite score was calculated as the mean of the normalized nonmotor test scores. Domain-specific composite scores were calculated as the mean of the normalized tests assigned to each domain.

Normalized scores were generated using a regression-based approach derived from a subset of PWoH without cognitive impairment. The normalization models were stratified by sex and adjusted for age, education, literacy, and study site. Timed tests, including Color Trails 1, Color Trails 2, timed gait, and dominant- and non-dominant-hand grooved pegboard tests, were reverse-coded so that higher normalized scores consistently represented better performance. For the present analysis, all normalized outcomes were multiplied by – 1 so that higher values consistently indicated poorer neurocognitive performance. Accordingly, a positive b coefficient represents poorer performance associated with CAD or predicted ASCVD risk.

### Covariates and other characteristics of study participants

Sociodemographic, behavioral, and cardiometabolic characteristics were obtained using standardized parent-study procedures. Age was recorded in years, and educational attainment was categorized according to the highest level completed. Smoking and alcohol use were classified as never, past, or current use. Physical activity was assessed from the reported frequency and duration of walking and moderate- and vigorous-intensity activity during a typical week, summarized as metabolic-equivalent minutes per week (22). Body-mass index was calculated as weight in kilograms divided by height in meters squared. The parent study also profiled systolic and diastolic blood pressure and laboratory measures, including glycated hemoglobin and lipid levels.

Ten-year predicted atherosclerotic cardiovascular disease (ASCVD) risk was estimated using the American College of Cardiology/American Heart Association Pooled Cohort Equations. The score incorporates age, sex, race, total cholesterol, high-density lipoprotein cholesterol, systolic blood pressure, antihypertensive treatment, diabetes status, and smoking status (23). In analyses, ASCVD risk was categorized as greater than 5% versus 5% or lower, consistent with the highest-risk category used in the parent CAD study (16).

### Statistical analysis

Participant characteristics were summarized using means with standard deviations (for normally-distributed) or medians with interquartile ranges (for skewed variables) for continuous variables, and frequencies with percentages for categorical variables. Analyses were conducted using complete-case data for each outcome-specific model; missing values were not imputed.

The primary exposure was imaging-defined CAD. The primary outcome was the global cognitive composite. Secondary outcomes were domain-level composite scores, and individual neurocognitive test scores. All neurocognitive outcomes were standardized and oriented so that higher scores indicated poorer performance; therefore, regression coefficients are interpretable as adjusted differences in standard-deviation units.

We used linear regression models to estimate associations between CAD and neurocognitive outcomes. Overall CAD models were adjusted for age, sex, and HIV status. Because HIV status was central to the cohort design and may influence neurocognitive performance, we also conducted HIV-stratified analyses among PWH and PWOH, with stratum-specific CAD models adjusted for age and sex. Formal CAD-by-HIV interaction terms were fitted in the overall cohort to assess whether CAD–cognition associations differed by HIV status.

As an alternative cardiovascular-risk exposure, we examined predicted 10-year ASCVD risk > 5%. Overall ASCVD-risk models were adjusted for HIV status only because age and sex are components of the ASCVD risk score. We also conducted exploratory HIV-stratified analyses of ASCVD risk > 5%; these stratum-specific models were not additionally adjusted for age or sex because these variables are incorporated into the ASCVD risk score. ASCVD-risk-by-HIV interaction terms were fitted in the overall cohort to assess whether associations differed by HIV status.

To account for multiple comparisons, we used the Benjamini-Hochberg false-discovery-rate (FDR) procedure within prespecified outcome tiers and model families(24). The global cognitive composite was treated as the single primary outcome. FDR correction was applied across domain-level composites for secondary analyses and across individual neurocognitive tests for exploratory analyses. We report nominal P values and FDR-adjusted q values, with q < 0.05 considered evidence of statistical significance after correction. Participants with missing data for a specific outcome or covariate were excluded from that model only. Statistical analyses were performed using R version 4.3.1 (R Foundation for Statistical Computing, Vienna, Austria), with heteroskedasticity-consistent robust standard errors.

## Results

### Analytic cohort

The analytic cohort included 512 older adults. The mean age was 57.9 years (SD, 6.3), 252 participants (49.2%) were female, 274 (53.5%) were PWOH, and 238 (46.5%) were PWH. Overall, 42 participants (8.2%) had imaging-defined CAD, and 203 (39.6%) had a predicted 10-year ASCVD risk score greater than 5% (Table 1).

In descriptive comparisons by HIV status, PWH were slightly younger than PWOH (57.2 vs 58.6 years; P = 0.012), while sex distribution, education, body-mass index, and smoking status were broadly similar between groups. Alcohol-use patterns differed by HIV status (P = 0.002), with past alcohol use more common among PWH. Predicted 10-year ASCVD risk scores differed by HIV status, with lower mean scores among PWH than PWOH (5.3% vs 6.7%; P = 0.027) and a smaller proportion of PWH classified as having ASCVD risk > 5% (32.8% vs 45.6%; P = 0.004) (Table 1).

Participants excluded from the primary multivariable CAD model were older, more likely to be living with HIV, had higher predicted ASCVD risk, and had lower educational attainment than those included, although few participants were excluded overall **(Supplementary Table S3).**

### CAD and neurocognitive performance in the overall cohort

In the primary analysis adjusted for age, sex, and HIV status, imaging-defined CAD was not associated with the global cognitive composite (b, 0.093 SD units; 95% CI, – 0.122 to 0.308; P = 0.397; within-tier FDR q = 0.397) (Fig. 1). In analyses of domain-level composite outcomes, CAD was not associated with attention and processing speed, learning and memory executive function and working memory, language or motor skills.

In individual-test analyses, CAD was associated with poorer Color Trails 2 performance, a task that reflects processing speed, visual attention, cognitive flexibility, and set shifting (b, 0.502 SD units; 95% CI, 0.184 to 0.821; P = 0.002; within-tier FDR q = 0.026). CAD was nominally associated with poorer Digits Forward performance, a measure of attention and immediate verbal span (b, 0.386; 95% CI, 0.028 to 0.744; P = 0.035), but this association did not remain significant after within-tier FDR correction (q = 0.210). Other test-level outcomes, overall domain-level outcomes, and global cognitive performance were not associated with CAD (Fig. 1).

### Predicted ASCVD risk and neurocognitive performance

In analyses adjusted for HIV status, predicted 10-year ASCVD risk greater than 5% was not associated with the global cognitive composite (b = 0.049 SD units; 95% CI, – 0.060 to 0.158; P = 0.379; within-tier FDR q = 0.379) (Fig. 2).

In domain-level analyses, ASCVD risk greater than 5% was nominally associated with poorer learning- and-memory domain performance (b = 0.188; 95% CI, 0.026 to 0.350; P = 0.023), but this association did not remain significant after within-tier FDR correction (q = 0.116). ASCVD risk was not associated with attention and processing speed, executive function and working memory, language, or motor skills after correction for multiple comparisons (Fig. 2).

In individual-test analyses, ASCVD risk greater than 5% was nominally associated with poorer WHO/UCLA Auditory Verbal Learning Test total-recall performance (b = 0.231; 95% CI, 0.048 to 0.414; P = 0.014), but this association did not survive within-tier FDR correction (q = 0.162). No other individual-test level, domain-level, or global cognitive outcomes were associated with predicted ASCVD risk (Fig. 2).

### HIV-stratified analyses

At individual test-level analyses stratified by HIV status, CAD was not associated with the global cognitive composite among either PWOH (b = 0.016; 95% CI, – 0.266 to 0.298; P = 0.910; q = 0.910) or PWH (b = 0.150; 95% CI, – 0.180 to 0.479; P = 0.375; q = 0.375) (Fig. 3). Among PWH, CAD was nominally associated with poorer Color Trails 2 performance (b = 0.646; 95% CI, 0.159 to 1.133; P = 0.010), but this association did not remain significant after within-tier FDR correction (q = 0.121). CAD was not associated with the global cognitive composite, domain-level composites, or other individual-test outcomes after FDR correction among PWH (Fig. 3A).

Among PWOH, CAD was nominally associated with poorer Digits Forward performance (b = 0.552; 95% CI, 0.163 to 0.940; P = 0.006), but this association did not meet the within-tier FDR threshold (q = 0.070). CAD was not associated with domain-level composites or other individual-test outcomes after FDR correction among PWOH (Fig. 3B).

Formal CAD-by-HIV interaction testing did not provide evidence that HIV status modified these associations, including for Digits Forward (P = 0.354) and Color Trails 2 (P = 0.575) (**Supplementary Table S2**).

## Discussion

In this cohort of older adults with and without HIV in southwestern Uganda, imaging-defined CAD was associated with poorer performance on tests of executive function. The estimated associations were small to moderate in magnitude when translated into effect sizes. Higher predicted 10-year ASCVD risk was also nominally associated with poorer verbal learning and memory performance, but neither remained significant after FDR correction. In contrast, CAD was not associated with poorer global cognitive performance or with domain-level performance.

Test-level associations between CAD and cognitive function were observed for Digits Forward and Color Trails; the test-specific pattern was most evident for Color Trails 2, with a weaker nominal signal for Digits Forward. Color Trails 2 places substantial demands on set-shifting, cognitive flexibility, visual attention, and psychomotor speed. This pattern is biologically plausible because CAD is a marker of vascular disease burden, characterized by endothelial dysfunction, inflammation, and microvascular injury (25). These pathophysiologic mechanisms can similarly disrupt frontal–subcortical networks supporting attention and executive function (2, 4, 26). Higher coronary artery calcium has likewise been associated with poorer cognitive performance, greater white-matter lesion burden, and reduced brain volumes (27, 28).

Our finding that CAD was not associated with the global cognitive composite is notable. Prior studies and reviews have linked cardiac disease, coronary atherosclerosis, and cardiovascular risk factors to worse cognition, but the reported effects are heterogeneous and vary by population, cognitive outcome, CAD definition, and severity of vascular disease (2, 26-28). The absence of an association between CAD and global cognition may partly reflect the low coronary disease burden in this cohort. Most participants had no detectable coronary calcium or angiographic disease, and nearly all identified abnormalities were mild; only three participants had an Agatston score ≥ 100. Predominantly low-burden, imaging-detected CAD may be insufficient to produce broad cognitive differences and also reduces power to assess dose–response relationships. In addition, global composites may dilute subtle effects concentrated in selected cognitive processes. Early or subcortical vascular injury typically affects attention, processing speed, executive control, and verbal learning efficiency or retrieval, whereas a predominantly amnestic storage profile is less common except with strategic lesions involving medial temporal or related memory structures (29-31).

In HIV-stratified analyses, CAD was nominally associated with poorer Digits Forward performance, reflecting attention and immediate auditory span, among PWOH, and poorer Color Trails 2 performance, reflecting executive function and set-shifting, among PWH; however, neither survived FDR correction, and interaction tests did not support effect modification. Therefore, the stratum-specific findings should not be taken as evidence of distinct CAD–cognition patterns in PWH and PWOH. This finding is notable because earlier pre-ART and early-ART literature reported substantial HIV-related cognitive vulnerability, including HIV-associated neurocognitive disorders and frontostriatal cognitive patterns (32, 33). However, more recent ART-era literature suggests that severe HIV-associated dementia has declined and that cognitive outcomes among treated PWH are increasingly shaped by ageing, vascular risk, and other comorbidities (12, 34, 35). Our findings are also consistent with recent data from Uganda, in which older PWH well-treated on ART had cognitive performance broadly comparable to demographically matched PWOH(36).

The ASCVD-risk models provide a complementary perspective. Unlike direct measurement of CAD, ASCVD risk scores compile data on age, CVD risk factors (e.g., cholesterol, hypertension, diabetes, smoking), which may be risk factors for vascular disease even in the absence of CAD. A predicted 10-year ASCVD risk score > 5% was nominally associated with poorer verbal learning and memory performance, but not with global cognition. These exploratory signals are consistent with the possibility that cumulative vascular risk reflects broader cerebrovascular vulnerability, including small-vessel and neurovascular-unit injury (25, 37, 38). Similar associations between cardiovascular-risk scores and subsequent cognitive decline have been reported in other ageing populations, including older PWH (38-41). These findings support further evaluation of simple cardiovascular-risk assessment to identify older adults who may benefit from intensified vascular-risk management and targeted cognitive monitoring.

Our findings have implications for ageing HIV and non-communicable disease (NCD) care in sub-Saharan Africa. CAD was not associated with poorer global cognitive performance, but its association with selected attention and executive-function tasks suggests that brief multidomain screening tools may not capture subtle deficits in visual attention, processing speed, cognitive flexibility, set-shifting, and immediate auditory attention. These functions may influence medication use, appointment adherence, and ability to complete other activities of daily living (42, 43). Older adults with HIV and known vascular disease or higher cardiovascular risk may benefit from cognitive function screening. For those with deficits, follow-up within integrated HIV–NCD care may provide benefit. The results do not support routine coronary computed tomography or extensive neuropsychological testing in resource-limited clinics; rather, they justify further research into the benefits of targeted cognitive screening.

This study has several limitations. CAD and neurocognitive performance were assessed at single time points, precluding causal inference and evaluation of cognitive change over time. In addition, coronary imaging and neurocognitive testing were not always concurrent and could be separated by up to 12 months. Although coronary atherosclerosis is unlikely to change substantially over such a period, cognitive performance and intervening health events may have changed, potentially attenuating or otherwise distorting the observed associations. A key limitation concerns the definition and severity of CAD. Although classified as imaging-defined CAD, detected disease was almost exclusively low burden and non-obstructive: positive CAD-RADS findings were limited to grades 1–2, no participant had grades 3–5, and only three had an Agatston score ≥ 100. The study therefore provides evidence mainly about mild imaging-detected CAD rather than advanced, obstructive, or symptomatic CAD. This narrow exposure range may have attenuated observable differences, prevented assessment of severity gradients, and limits generalizability to patients with clinically manifest disease. Although the primary CAD models adjusted for age, sex, and HIV status, and ASCVD-risk models adjusted for HIV status only, residual confounding from unmeasured vascular, socioeconomic, and life-course exposures remains possible. In addition, the study population was drawn from parent cohorts in southwestern Uganda, which may limit generalizability to other settings and to populations with more advanced symptomatic CAD. Finally, complete-case modelling may have biased associations toward the null because participants excluded from multivariable models appeared to have higher vascular and cognitive-risk profiles, including older age, higher ASCVD risk, greater representation of PWH, and lower educational attainment.

The study also has notable strengths. CAD was assessed using imaging-based measures, including CAD-RADS and Agatston calcium score, rather than self-reported cardiovascular history alone. Neurocognitive performance was examined at global, domain, and individual-test levels, allowing more granular characterization of cognitive vulnerability. The inclusion of people with and without HIV within the same analytic framework is especially relevant in settings where HIV and cardiometabolic disease increasingly overlap. Finally, the study addresses an important evidence gap, as most research on CAD-related cognitive vulnerability has been conducted in high-income settings.

## Conclusion

In this cohort of older adults with and without HIV in southwestern Uganda, Imaging-defined CAD was associated with poorer Color Trails 2 performance, reflecting executive function and processing speed. Higher predicted 10-year ASCVD risk showed nominal associations with verbal learning and memory measures, but these did not remain significant after FDR correction. In contrast, CAD was not associated with poorer global cognitive performance or with domain-level performance. These findings suggest that CAD and elevated cardiovascular risk may be linked to selective cognitive vulnerabilities rather than generalized cognitive impairment. Larger longitudinal studies, spanning a broader range of coronary disease severity, are needed to corroborate these associations, and determine whether cardiovascular risk reduction can help preserve cognitive function in ageing African populations.

## Supplementary Material

This is a list of supplementary files associated with this preprint. Click to download.

• SupplementaryTableS1-S3.docx

## Figures and Tables

**Figure 1:**
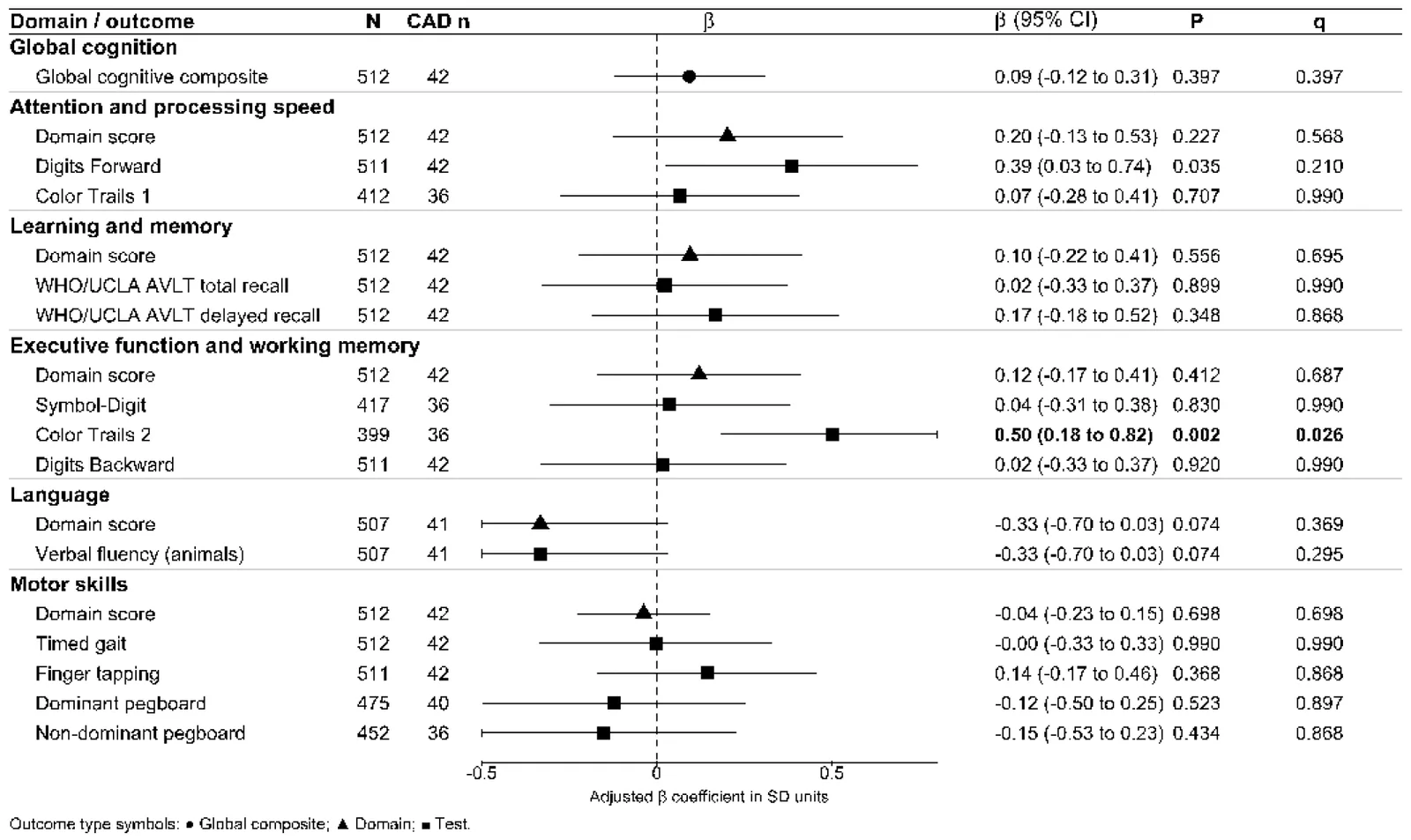
Association between imaging-defined coronary artery disease and neurocognitive performance among older adults with and without HIV in southwestern Uganda, in the overall cohort. CAD denotes coronary artery disease; CI, confidence interval; and HIV, human immunodeficiency virus.

**Figure 2:**
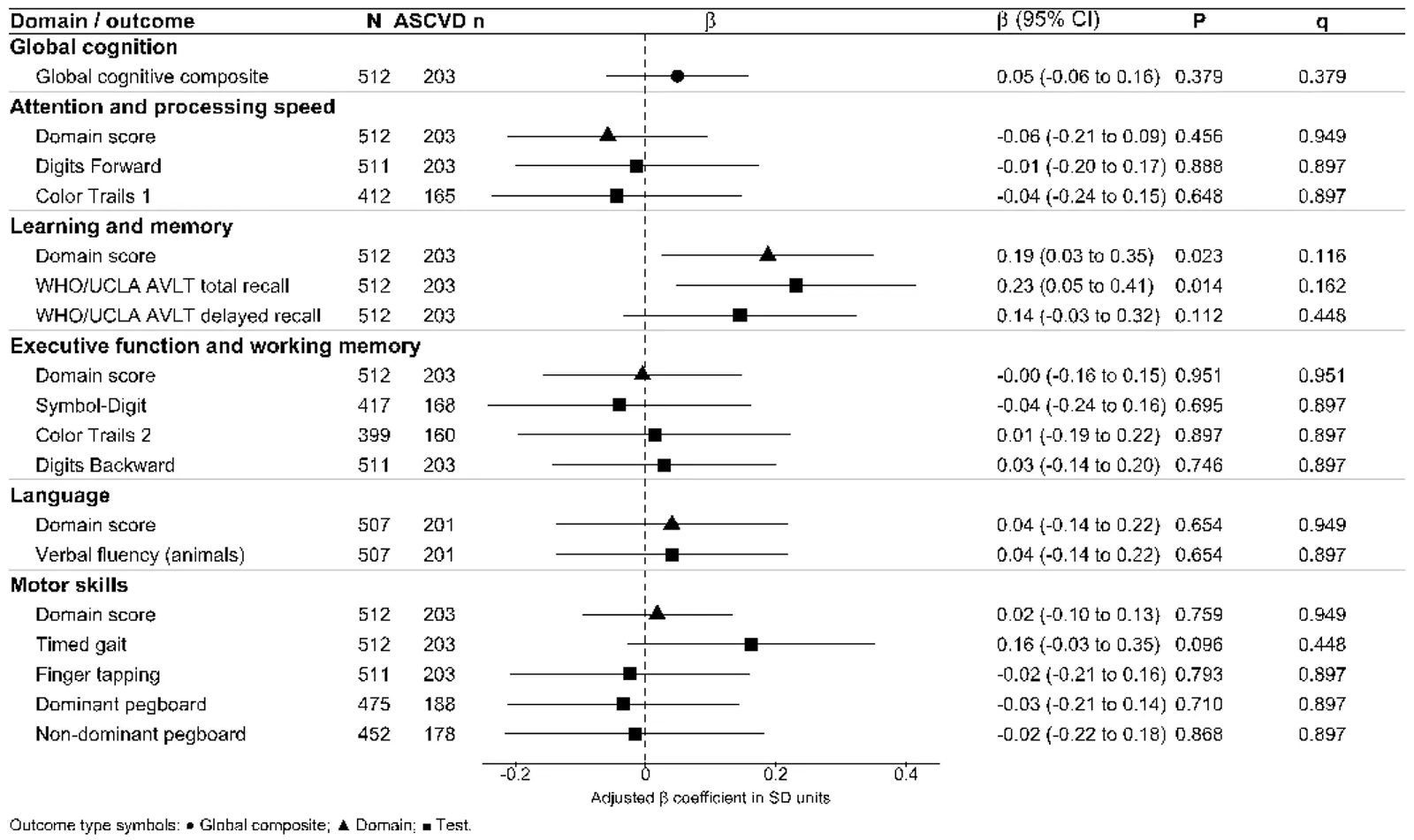
Association between predicted 10-year atherosclerotic cardiovascular disease (ASCVD) risk greater than 5% and neurocognitive performance among older adults with and without HIV in southwestern Uganda. Positive beta coefficients indicate poorer performance. AVLT, Auditory Verbal Learning Test; CI, confidence interval; UCLA, University of California, Los Angeles; WHO, World Health Organization.

**Figure 3:**
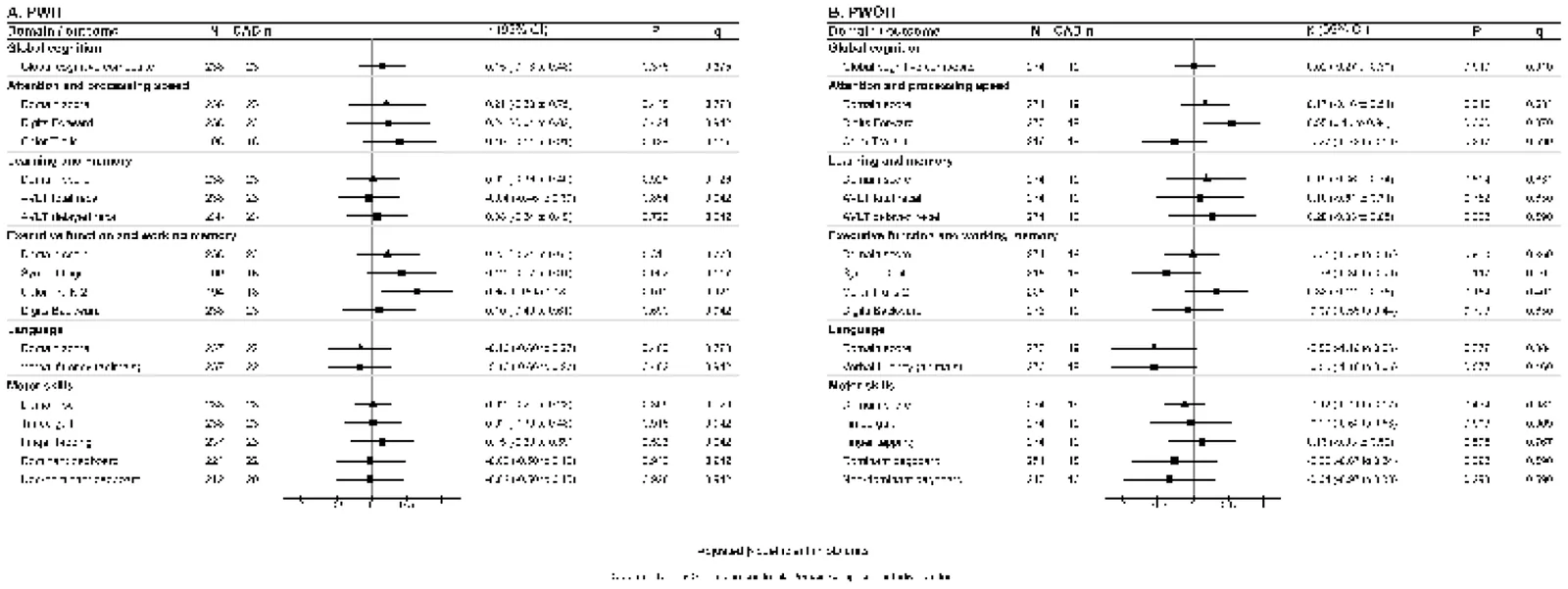
Association between imaging-defined coronary artery disease and neurocognitive performance, stratified by HIV status in southwestern Uganda. Shown are b coefficients and 95% confidence intervals for the association between coronary artery disease (CAD) and neurocognitive performance, estimated separately among people without HIV (PWOH) and people with HIV (PWH).

**Table 1:** Sociodemographic, behavioral, cardiometabolic, and coronary imaging characteristics of the analytic cohort, overall and by HIV status

Characteristic	Overall cohort (N = 512)	PWOH (n = 274)	PWH (n = 238)	P value
Sociodemographic characteristics				
Age, years	57.9 ± 6.3	58.6 ± 6.7	57.2 ± 5.8	0.012
Female sex, no. (%)	252 (49.2)	133 (48.5)	119 (50.0)	0.790
Education level, no. (%)				0.227
No formal education	75 (14.6)	47 (17.2)	28 (11.8)	—
Primary school, P1–P6	186 (36.3)	105 (38.3)	81 (34.0)	—
Primary school, P7	116 (22.7)	57 (20.8)	59 (24.8)	—
Secondary school, S1–S4	76 (14.8)	37 (13.5)	39 (16.4)	—
S5 or above	59 (11.5)	28 (10.2)	31 (13.0)	—
Behavioral characteristics				
Smoking status, no. (%)				0.181
Never smoker	297 (58.0)	153 (55.8)	144 (60.5)	—
Past smoker	173 (33.8)	93 (33.9)	80 (33.6)	—
Current smoker	42 (8.2)	28 (10.2)	14 (5.9)	—
Alcohol use status, no. (%)				0.002
Never drinker	156 (30.5)	98 (35.8)	58 (24.4)	—
Past drinker	203 (39.6)	90 (32.8)	113 (47.5)	—
Current drinker	153 (29.9)	86 (31.4)	67 (28.2)	—
Cardiometabolic characteristics				
Body-mass index, kg/m^2^	24.2 ± 5.1	24.6 ± 5.5	23.8 ± 4.5	0.095
Systolic blood pressure, mm Hg	124.1 ± 19.4	126.1 ± 19.9	121.8 ± 18.6	0.013
Diastolic blood pressure, mm Hg	78.5 ± 11.7	78.8 ± 11.3	78.2 ± 12.2	0.536
Glycated hemoglobin, %	5.8 ± 1.2	5.8 ± 1.0	5.8 ± 1.3	0.944
Total cholesterol, mmol/L	4.2 ± 1.0	4.3 ± 0.9	4.1 ± 1.0	0.073
LDL cholesterol, mmol/L	2.1 ± 0.9	2.2 ± 0.9	2.1 ± 0.8	0.154
Sociodemographic characteristics				
HDL cholesterol, mmol/L	1.3 ± 0.5	1.3 ± 0.4	1.3 ± 0.6	0.584
Predicted 10-year ASCVD risk, %	6.1 ± 7.3	6.7 ± 8.0	5.3 ± 6.4	0.027
Predicted 10-year ASCVD risk, no. (%)				0.004
≤5%	309 (60.4)	149 (54.4)	160 (67.2)	—
>5%	203 (39.6)	125 (45.6)	78 (32.8)	—
Coronary imaging characteristics				
CAD* present, no. (%)	42 (8.2)	19 (6.9)	23 (9.7)	0.333
CAD-RADS score, no. (%)				0.204
0	449 (93.2)	248 (94.7)	201 (91.4)	—
1–2	33 (6.8)	14 (5.3)	19 (8.6)	—
Agatston calcium score, no. (%)				0.153
0	483 (94.3)	260 (94.9)	223 (93.7)	—
1–99	26 (5.1)	11 (4.0)	15 (6.3)	—
≥100	3 (0.6)	3 (1.1)	0 (0.0)	—

* CAD was defined as the presence of calcified or non-calcified plaque on coronary computed tomography angiography or an Agatston coronary artery calcium score greater than 0. ASCVD denotes atherosclerotic cardiovascular disease; CAD, coronary artery disease; CAD-RADS, Coronary Artery Disease–Reporting and Data System; HDL, high-density lipoprotein; HIV, human immunodeficiency virus; LDL, low-density lipoprotein; PWH, people with HIV; and PWOH, people without HIV.

## Data Availability

The datasets analyzed for this manuscript are not publicly available because they contain sensitive health information, including HIV status, cardiovascular measurements, neurocognitive performance data, and behavioral characteristics, even though the analytic files were de-identified. Data may be made available upon reasonable request to the corresponding author and with permission from the relevant parent study investigators, institutional data custodians, and ethics/regulatory authorities, subject to applicable data-sharing agreements.

## Abbreviations

ACC/AHA: American College of Cardiology/American Heart Association
ASCVD: atherosclerotic cardiovascular disease
AVLT: Auditory Verbal Learning Test
BMI: body-mass index
CAC: coronary artery calcium
CAD: coronary artery disease
CAD-RADS: Coronary Artery Disease–Reporting and Data System
CCTA: coronary computed tomography angiography
CI: confidence interval
CT: computed tomography
CVD: cardiovascular disease
HbA1c: glycated hemoglobin
HDL: high-density lipoprotein
HIV: human immunodeficiency virus
IQR: interquartile range
LDL: low-density lipoprotein
MET: metabolic equivalent of task
NCD: noncommunicable disease
PCE: Pooled Cohort Equations
PWH: people with HIV
PWOH: people without HIV
SD: standard deviation
SE: standard error
UCLA: University of California,Los Angeles
WHO: World Health Organization

## References

[1] Deckers K, Schievink SH, Rodriquez MM, van Oostenbrugge RJ, van Boxtel MP, Verhey FR, Coronary heart disease and risk for cognitive impairment or dementia: Systematic review and meta-analysis. PLoS ONE. 2017;12(9):e0184244. 10.1371/journal.pone.0184244 PMC5590905 28886155

[2] Burkauskas J, Lang P, Bunevičius A, Neverauskas J, Bučiūtė-Jankauskienė M, Mickuvienė N. Cognitive function in patients with coronary artery disease: A literature review. J Int Med Res. 2018;46(10):4019–31. 10.1177/0300060517751452 PMC6166352 30157691

[3] Mejia-Renteria H, Travieso A, Matías-Guiu JA, Yus M, Espejo-Paeres C, Finocchiaro F, Coronary microvascular dysfunction is associated with impaired cognitive function: the Cerebral-Coronary Connection study (C3 study). Eur Heart J. 2023;44(2):113–25. 10.1093/eurheartj/ehac521 PMC9825810 36337036

[4] Wang Y, Zhang H, Liu L, Li Z, Zhou Y, Wei J, Cognitive function and cardiovascular health in the elderly: network analysis based on hypertension, diabetes, cerebrovascular disease, and coronary heart disease. Front Aging Neurosci. 2023;15:1229559. 10.3389/fnagi.2023.1229559 PMC10436622 37600511

[5] Mworozi K, Ameda F, Byanyima RK, Nakasujja N. Carotid artery plaque detected on ultrasound is associated with impaired cognitive state in the elderly: a population-based study in Wakiso district, Uganda. J Clin Neurosci. 2019;68:194–200. 10.1016/j.jocn.2019.06.011 31301929

[6] Nichols E, Steinmetz JD, Vollset SE, Fukutaki K, Chalek J, Abd-Allah F, Estimation of the global prevalence of dementia in 2019 and forecasted prevalence in 2050: an analysis for the Global Burden of Disease Study 2019. Lancet Public Health. 2022;7(2):e105–25. 10.1016/S2468-2667(21)00249-8 PMC8810394 34998485

[7] Akinyemi RO, Yaria J, Ojagbemi A, Guerchet M, Okubadejo N, Njamnshi AK, Dementia in Africa: Current evidence, knowledge gaps, and future directions. Alzheimer's Dement J Alzheimer's Assoc. 2022;18(4):790–809. 10.1002/alz.12432 PMC8957626 34569714

[8] McCutcheon K, Nqebelele U, Murray L, Thomas TS, Mpanya D, Tsabedze N. Cardiac and renal comorbidities in aging people living with HIV. Circul Res. 2024;134(11):1636–60. 10.1161/CIRCRESAHA.124.323948 PMC11122746 38781295

[9] Yitbarek GY, Ayehu GW, Ayele BA, Bayih WA, Gebremariam AD, Tiruneh SA. Cognitive impairment and its associated factors among HIV/AIDS patients on anti retro-viral therapy in Sub-Saharan Africa: systematic review and meta-analysis. Neurol Psychiatry Brain Res. 2020;38:83–91.

[10] Shah AS, Stelzle D, Lee KK, Beck EJ, Alam S, Clifford S, Global burden of atherosclerotic cardiovascular disease in people living with HIV: systematic review and meta-analysis. Circulation. 2018;138(11):1100–12. 10.1161/CIRCULATIONAHA.117.033369 PMC6221183 29967196

[11] So-Armah K, Benjamin LA, Bloomfield GS, Feinstein MJ, Hsue P, Njuguna B, HIV and cardiovascular disease. lancet HIV. 2020;7(4):e279–93. 10.1016/S2352-3018(20)30036-9 PMC9346572 32243826

[12] McIntosh EC, Tureson K, Rotblatt LJ, Singer EJ, Thames AD. HIV, vascular risk factors, and cognition in the combination antiretroviral therapy era: a systematic review and meta-analysis. J Int Neuropsychol Soc. 2021;27(4):365–81. 10.1017/S1355617720001022 PMC9618305 33161930

[13] Olajide T, Ogungbemi E, Olajide G, Ogundijo D, Osakuade O, Moshood F. HIV-associated neurocognitive disorders in Africa: challenges, peculiarities, and future directions. AIDS Res Therapy. 2024;21(1):88. 10.1186/s12981-024-00677-6 PMC11605981 39609893

[14] Zenebe Y, Necho M, Yimam W, Akele B. Worldwide occurrence of HIV-associated neurocognitive disorders and its associated factors: a systematic review and meta-analysis. Front Psychiatry. 2022;13:814362. 10.3389/fpsyt.2022.814362 PMC9193596 35711575

[15] Longenecker CT, Bogorodskaya M, Margevicius S, Nazzinda R, Bittencourt MS, Erem G, Sex modifies the association between HIV and coronary artery disease among older adults in Uganda. J Int AIDS Soc. 2022;25(1):e25868. 10.1002/jia2.25868 PMC8741262 34995413

[16] Siedner MJ, Ghoshhajra B, Erem G, Nassanga R, Randhawa M, Ochieng A, Epidemiology of coronary atherosclerosis among people living with HIV in Uganda: a cross-sectional study. Ann Intern Med. 2025;178(4):468–78. 10.7326/ANNALS-24-02233 PMC12022967 40073231

[17] Asiimwe S, Acan M, Maling S, Nakasujja N, Atwine F, Tindimwebwa E The Uganda Aging Cohort Study: Current Lessons and Future Opportunities. Alzheimer's & Dementia. 2025;21:e100054.

[18] Cury RC, Leipsic J, Abbara S, Achenbach S, Berman D, Bittencourt M CAD-RADS^™^ 2.0–2022 coronary artery disease–Reporting and data system an expert consensus document of the Society of Cardiovascular Computed Tomography (SCCT), the American College of Cardiology (ACC), the American College of Radiology (ACR) and the North America Society of Cardiovascular Imaging (NASCI). Radiology: Cardiothoracic Imaging. 2022;4(5):e220183. 10.1148/ryct.220183 PMC9627235 36339062

[19] Robertson KR, Nakasujja N, Wong M, Musisi S, Katabira E, Parsons TD, Pattern of neuropsychological performance among HIV positive patients in Uganda. BMC Neurol. 2007;7(1):8. 10.1186/1471-2377-7-8 PMC1855062 17411437

[20] Sacktor N, Nakasujja N, Okonkwo O, Skolasky RL, Robertson K, Musisi S, Longitudinal neuropsychological test performance among HIV seropositive individuals in Uganda. J Neurovirol. 2013;19(1):48–56. 10.1007/s13365-012-0139-5 PMC3568232 23315423

[21] Maj M, D'Elia L, Satz P, Janssen R, Zaudig M, Uchiyama C, Evaluation of two new neuropsychological tests designed to minimize cultural bias in the assessment of HIV-1 seropositive persons: a WHO study. Arch Clin Neuropsychol. 1993;8(2):123–35. 14589670

[22] Mendes MA, Da Silva I, Ramires V, Reichert F, Martins R, Ferreira R, Metabolic equivalent of task (METs) thresholds as an indicator of physical activity intensity. PLoS ONE. 2018;13(7):e0200701. 10.1371/journal.pone.0200701 PMC6053180 30024953

[23] Rout A, Duhan S, Umer M, Li M, Kalra D. Atherosclerotic cardiovascular disease risk prediction: current state-of-the-art. Heart. 2024;110(15):1005–14. 10.1136/heartjnl-2023-322928 37918900

[24] Benjamini Y, Hochberg Y. Controlling the false discovery rate: a practical and powerful approach to multiple testing. J Roy Stat Soc: Ser B (Methodol). 1995;57(1):289–300.

[25] Gorelick PB, Scuteri A, Black SE, DeCarli C, Greenberg SM, Iadecola C Vascular contributions to cognitive impairment and dementia: a statement for healthcare professionals from the American Heart Association/American Stroke Association. stroke. 2011;42(9):2672–713. 10.1161/STR.0b013e3182299496 PMC3778669 21778438

[26] Eggermont LH, De Boer K, Muller M, Jaschke AC, Kamp O, Scherder EJ. Cardiac disease and cognitive impairment: a systematic review. Heart. 2012;98(18):1334–40. 10.1136/heartjnl-2012-301682 22689718

[27] Vidal J-S, Sigurdsson S, Jonsdottir MK, Eiriksdottir G, Thorgeirsson G, Kjartansson O, Coronary artery calcium, brain function and structure: the AGES-Reykjavik Study. Stroke. 2010;41(5):891–7. 10.1161/STROKEAHA.110.579581 PMC3298743 20360538

[28] Xia C, Vonder M, Sidorenkov G, Oudkerk M, de Groot JC, van der Harst P, The relationship of coronary artery calcium and clinical coronary artery disease with cognitive function: a systematic review and meta-analysis. J Atheroscler Thromb. 2020;27(9):934–58. 10.5551/jat.52928 PMC7508729 32062643

[29] Markus HS, Joutel A. The pathogenesis of cerebral small vessel disease and vascular cognitive impairment. Physiol Rev. 2025;105(3):1075–171. 10.1152/physrev.00028.2024 PMC12182829 39965059

[30] Jokinen H, Laakso HM, Arola A, Paajanen TI, Virkkala J, Särkämö T, Executive functions and processing speed in covert cerebral small vessel disease. Eur J Neurol. 2025;32(1):e16533. 10.1111/ene.16533 PMC11622512 39475227

[31] Barbosa BJAP, Siqueira Neto JI, Alves GS, Sudo FK, Suemoto CK, Tovar-Moll F, Diagnosis of vascular cognitive impairment: recommendations of the scientific department of cognitive neurology and aging of the Brazilian Academy of Neurology. Dement Neuropsychologia. 2022;16:53–72. 10.1590/1980-5764-DN-2022-S104PT PMC9746000 36533159

[32] Antinori A, Arendt G, Becker J, Brew B, Byrd D, Cherner M, Updated research nosology for HIV-associated neurocognitive disorders. Neurology. 2007;69(18):1789–99. 10.1212/01.WNL.0000287431.88658.8b PMC4472366 17914061

[33] Heaton R, Clifford D, Franklin D Jr, Woods S, Ake C, Vaida F, HIV-associated neurocognitive disorders persist in the era of potent antiretroviral therapy: CHARTER Study. Neurology. 2010;75(23):2087–96. 10.1212/WNL.0b013e318200d727 PMC2995535 21135382

[34] Deng L, Zhang X, Gao Y, Turner D, Qian F, Lu H, Association of HIV infection and cognitive impairment in older adults: a meta-analysis. Ageing Res Rev. 2021;68:101310. 10.1016/j.arr.2021.101310 PMC10767715 33640473

[35] Wang Y, Liu M, Lu Q, Farrell M, Lappin JM, Shi J, Global prevalence and burden of HIV-associated neurocognitive disorder: a meta-analysis. Neurology. 2020;95(19):e2610–21. 10.1212/WNL.0000000000010752 32887786

[36] Tanner JA, Valdespino R, Kautz TF, Masette G, Hromas G, Wang CP, Chronic HIV Infection Association with Cognition and ADRD Plasma Biomarkers in Uganda. Alzheimer's Dement. 2025;21:e098870.

[37] Smith EE, Aparicio HJ, Gottesman RF, Goyal MS, Greenberg SM, Schneider JA, Vascular contributions to cognitive impairment and dementia in the United States: prevalence and incidence: a scientific statement from the American Heart Association. Stroke. 2025;56(10):e317–30. 10.1161/STR.0000000000000494 40820756

[38] Jia R, Wang Q, Huang H, Yang Y, Chung YF, Liang T. Cardiovascular disease risk models and dementia or cognitive decline: a systematic review. Front Aging Neurosci. 2023;15:1257367. 10.3389/fnagi.2023.1257367 PMC10613491 37904838

[39] Kaffashian S, Dugravot A, Nabi H, Batty GD, Brunner E, Kivimäki M, Predictive utility of the Framingham general cardiovascular disease risk profile for cognitive function: evidence from the Whitehall II study. Eur Heart J. 2011;32(18):2326–32. 10.1093/eurheartj/ehr133 PMC3172575 21606085

[40] Chow FC, Lyass A, Mahoney TF, Massaro JM, Triant VA, Wu K, Baseline 10-year cardiovascular risk scores predict cognitive function in older persons, and particularly women, living with human immunodeficiency virus infection. Clin Infect Dis. 2020;71(12):3079–85. 10.1093/cid/ciz1214 PMC7819524 31899478

[41] Schaich CL, Yeboah J, Espeland MA, Baker LD, Ding J, Hayden KM, Association of vascular risk scores and cognitive performance in a diverse cohort: the multi-ethnic study of atherosclerosis. Journals Gerontology: Ser A. 2022;77(6):1208–15. 10.1093/gerona/glab189 PMC9159669 34216214

[42] Insel K, Morrow D, Brewer B, Figueredo A. Executive function, working memory, and medication adherence among older adults. Journals Gerontol Ser B: Psychol Sci Social Sci. 2006;61(2):P102–7. 10.1093/geronb/61.2.p102 16497953

[43] Ettenhofer ML, Hinkin CH, Castellon SA, Durvasula R, Ullman J, Lam M, Aging, neurocognition, and medication adherence in HIV infection. Am J Geriatric Psychiatry. 2009;17(4):281–90. 10.1097/JGP.0b013e31819431bd PMC2679810 19307857

[R44] 44.Supplementary Table. S1. Neurocognitive assessments and normalized outcomes included in the analysis.

